# Characterization of TLX Expression in Neural Stem Cells and Progenitor Cells in Adult Brains

**DOI:** 10.1371/journal.pone.0043324

**Published:** 2012-08-30

**Authors:** Shengxiu Li, Guoqiang Sun, Kiyohito Murai, Peng Ye, Yanhong Shi

**Affiliations:** Department of Neurosciences, Cancer Center, Beckman Research Institute of City of Hope, Duarte, California, United States of America; Center for Regenerative Therapies Dresden, Germany

## Abstract

TLX has been shown to play an important role in regulating the self-renewal and proliferation of neural stem cells in adult brains. However, the cellular distribution of endogenous TLX protein in adult brains remains to be elucidated. In this study, we used immunostaining with a TLX-specific antibody to show that TLX is expressed in both neural stem cells and transit-amplifying neural progenitor cells in the subventricular zone (SVZ) of adult mouse brains. Then, using a double thymidine analog labeling approach, we showed that almost all of the self-renewing neural stem cells expressed TLX. Interestingly, most of the TLX-positive cells in the SVZ represented the thymidine analog-negative, relatively quiescent neural stem cell population. Using cell type markers and short-term BrdU labeling, we demonstrated that TLX was also expressed in the Mash1+ rapidly dividing type C cells. Furthermore, loss of TLX expression dramatically reduced BrdU label-retaining neural stem cells and the actively dividing neural progenitor cells in the SVZ, but substantially increased GFAP staining and extended GFAP processes. These results suggest that TLX is essential to maintain the self-renewing neural stem cells in the SVZ and that the GFAP+ cells in the SVZ lose neural stem cell property upon loss of TLX expression.Understanding the cellular distribution of TLX and its function in specific cell types may provide insights into the development of therapeutic tools for neurodegenerative diseases by targeting TLX in neural stem/progenitors cells.

## Introduction

Nuclear receptor TLX plays an important role in vertebrate brain functions [1]–[3]. We have shown that TLX is an essential regulator of adult neural stem cell self-renewal [3], through transcriptional repression of downstream target genes by complexing with histone-modifying enzymes [4]–[6], or by activating Wnt/β-catenin pathway [7]. TLX has also been shown to maintain adult hippocampal neural progenitor proliferation upon hypoxia by regulating Oct3/4 expression, and activates neuronal lineage commitment by inducing Mash1 expression [8]–[10]. TLX expression is regulated by microRNAs miR-9 and let-7 [11], [12]. In adult brains, the TLX-positive cells in the hippocampal dentate gyrus play an important role in learning and memory [13], whereas the TLX-expressing cells in the SVZ were shown to be slowly-dividing neural stem cells [14], [15]. TLX also plays a role in neural development by regulating neural stem cells of the developing brain [16]–[18]. However, due to the difficulty of TLX immunostaning in adult brains, data on endogenous TLX expression in adult brains are still lacking. The cellular identity of the TLX-expressing cells remains to be determined.

Neural stem cells in adult brains reside in the subgranular cell layer of the hippocampus and the SVZ [19]. The SVZ neural stem cells correspond to a rare population of relatively quiescent cells [20]. These type B cells serve as primary precursors and give rise to rapidly dividing type C cells. Type C cells then generate type A neuroblasts that differentiate into neurons destined to the olfactory bulbs [21].

Classical studies of neurogenesis used tritiated (^3^H) thymidine to mark cells undergoing DNA synthesis [22], [23]. The generation of antibodies specific for the thymidine analog bromodeoxyuridine (BrdU) eliminated the need to label dividing cells with radioactivity [24], [25] and advanced the field of neurogenesis study dramatically [26], [27]. In addition to BrdU, several recent reports have used iododeoxyuridine (IdU) and chlorodeoxyuridine (CIdU), thymidine analogs similar to BrdU, to label dividing cells [28]–[32].

In this study, TLX immunostaining is used to characterize the TLX-expressing cells in combination with thymidine analog labeling. We found that TLX was expressed in both the relatively quiescent neural stem cells and the rapidly dividing neural progenitor cells in the SVZ of adult mouse brains. Most of the TLX-positive cells in the SVZ were quiescent and did not incorporate any thymidine analogs. Moreover, we showed that TLX was expressed in a subpopulation of transit-amplifying type C cells. In TLX−/− brains we observed dramatically reduced BrdU-retaining neural stem cells and rapidly dividing neural progenitor cells. This finding is crucial for further understanding the role of TLX in neural stem/progenitor cells and in adult neurogenesis.

## Results

### TLX is expressed in both neural stem cells and rapidly dividing neural progenitor cells

We have shown that TLX is an essential regulator of adult neural stem cell population [3]. Using a β-galactosidase (β-gal) reporter, which was knocked into the endogenous TLX locus, we showed that TLX is highly expressed in the SVZ and the subgranular layer of the hippocampal dentate gyrus, the two well-characterized adult neurogenic areas, and displayed scattered distribution in the cortex [3]. The expression of TLX was further determined using a CreER^T2^ reporter under the control of the TLX gene regulatory sequences [14]. Although the expression of these reporter genes is informative about TLX expression, it does not necessarily represent the true status of endogenous TLX expression, since the half-life of the reporters may not be the same as the endogenous TLX protein.

**Figure 1:**
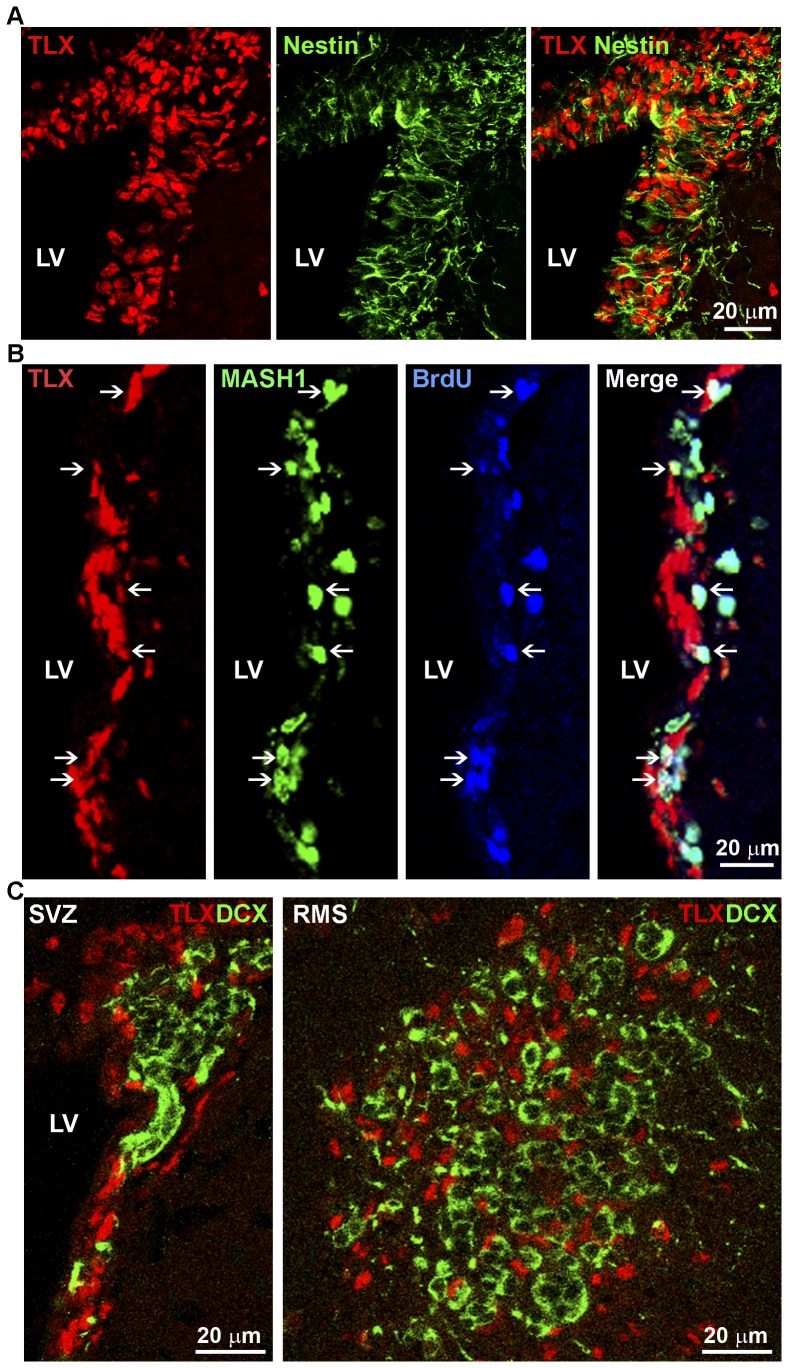
Co-staining of TLX cell type-specific markers in the SVZ. **A.** Co-staining of TLX with Nestin in the SVZ of mouse brains. **B.** Co-localization of TLX with Mash1 in the SVZ of mouse brains. **C.** Co-staining of TLX with DCX in both the SVZ and the rostral migratory stream (RMS). LV stands for the lateral ventricles. Scale bar, 20 µm for all panels. Examples of TLX and Mash1 double-positive cells were indicated by arrows.

To determine the expression pattern of endogenous TLX protein in the adult brain, we developed an immunohistochemistry protocol using a TLX-specific antibody [18]. TLX was highly expressed in the (SVZ) of adult mouse brains with nucleus-specific staining (Fig. 1A, B, C). Co-staining of TLX with cell type-specific markers revealed that neural stem cells that expressed nestin and GFAP in the SVZ frequently stained positive for TLX (Fig. 1A, Fig. S1). To determine if TLX was also expressed in the transit-amplifying neural progenitor cells, we co-stained TLX with the transit-amplifying type C cell marker Mash1. TLX was expressed in a fraction of Mash1+ cells (Fig. 1B, indicated by arrows, and Fig. S2) and we noticed that the TLX+Mash1+ cells were usually attached to the TLX+Mash1- cell clusters in the SVZ (Fig. 1B). These TLX+Mash1+ cells may represent early C cells that are presumably newly derived from the TLX+Mash1- type B cells. On the other hand, the TLX-Mash1+ cells are usually distant from the TLX+Mash1- cell clusters and may represent the late stage type C cells that have migrated away from the type B stem cell pool. The Mash1+ cells were also labeled by a 6 hr BrdU pulse (Fig. 1B), consistent with the notion that the 6 hr BrdU pulse labels the rapidly dividing cells. In contrast, TLX expression was not detected in the DCX+ type A neuroblasts in either the SVZ or the rostral migratory stream (RMS) (Fig. 1C). These results indicate that TLX is expressed in both type B neural stem cells and type C transit-amplifying neural progenitor cells.

**Figure 2:**
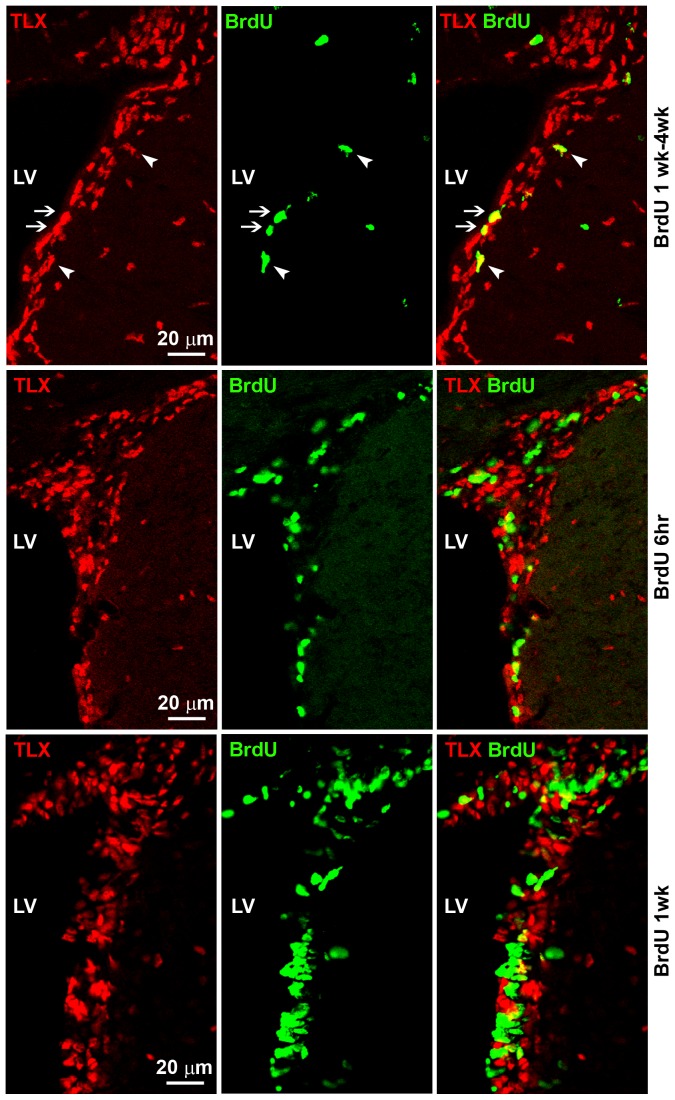
Co-staining of TLX and BrdU in the SVZ. Co-staining of TLX and BrdU in the SVZ of mice that were treated with BrdU for 1 week, followed by 4 week survival (long-term BrdU labeling), mice that were treated with BrdU once follwed by 6 hr survival (6 hr BrdU chase), or mice that were treated with BrdU once daily for 1 week (1 week BrdU treatment). LV stands for the lateral ventricles. Scale bar, 20 µm for all panels. Examples of TLX-positive, BrdU label-retaining cells that represent the ventricle-containing B1 cells were indicated by arrows, and examples of TLX-positive, BrdU label-retaining non-ventricle-containing B2 cells were indicated by arrowheads.

The adult mammalian SVZ contains relatively quiescent or slowly dividing neural stem cells. It has been shown that the slowly dividing, BrdU label-retaining cells represent neural stem cells in the adult brain [33]. To determine if the TLX-expressing cells are the BrdU label-retaining neural stem cells *in vivo*, we treated 6-week-old wild type mice with BrdU for 1 week and then allowed them to survive for 4 weeks (long-term BrdU labeling). Co-staining of TLX and BrdU revealed that 66±17% (n = 3) of the BrdU label-retaining cells were also TLX-positive (Fig. 2, top panels, and Fig. S3), suggesting that the TLX-positive cells make up the primary population of the BrdU label-retaining neural stem cells in the SVZ of adult brains.

The type B neural stem cells can be subdivided into the ventricle-contacting B1 cells and the non-ventricle-contacting B2 cells [21], [34]. Some of the TLX-positive, BrdU label-retaining cells faced the lateral wall of the lateral ventricles and therefore represent the ventricle-contacting B1 cells (arrow-pointed cells), whereas other TLX-positive and BrdU label-retaining cells localized away from the lateral ventricles (arrow head-pointed cells). These cells should represent the non-ventricle-contacting B2 cells (Fig. 2, top panels).

To further characterize the TLX-positive cells, we also used short-term BrdU labeling. BrdU was injected into adult mice once and the treated animals were sacrificed 6 hr after BrdU injection (6 hr BrdU pulse, Fig. 2, middle panels, and Fig. S3). This method mainly labels the rapidly dividing cells. In parallel, mice were injected with BrdU once daily for 7 days and animals were sacrificed the next day after the last BrdU treatment (1 week BrdU treatment, Fig. 2, lower panels). This method labels cells that undergo division over the 1 week period [35]. We found that 37±9% (n = 3) of the 6 hr BrdU pulse-labeled cells were TLX-positive, and 21±9% (n = 3) of the 1 week BrdU-labeled cells were TLX-positive (Fig. 2). Interestingly, in each of the BrdU-labeling schemes, the majority of TLX-positive cells were BrdU-negative (Fig. 2), suggesting that most of the TLX-positive cells represent the relatively quiescent stem cell population. A small fraction of the TLX-positive cells are the slowly dividing, BrdU label-retaining cells and a fraction of the TLX-positive cells are the actively dividing, short-term BrdU labeling cells.

### Most of the self-renewing neural stem cells are TLX-positive cells

**Figure 3:**
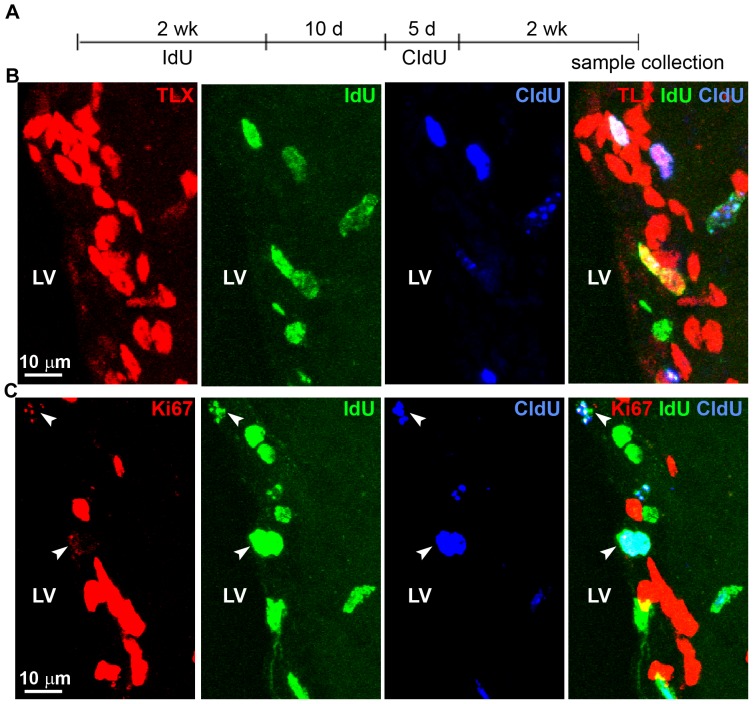
Co-labeling of TLX with IdU and CIdU in the SVZ. **A.** Schematics of IdU and CIdU treatment of adult mouse brains. **B.** Co-staining of TLX with IdU and CIdU in the SVZ of IdU and CIdU-treated mice. **C.** Co-staining of Ki67 with IdU and CIdU in the SVZ of IdU and CIdU-treated mice. Scale bar, 10 µm for all panels. The IdU, CIdU and Ki67 triple-positive cells were indicated by arrowheads.

To further determine the expression of TLX in the self-renewing neural stem cells, we took a double thymidine analog labeling strategy to monitor the expression of TLX in label-retaining cells that re-enter the cell cycle [36]. For this purpose, 6-week-old mice were treated with IdU in drinking water for 2 weeks, followed by 10 days of no treatment. These mice were then injected with CIdU once daily for 5 days, and sacrificed two weeks after the last CIdU injection, which is about 4 weeks from the end of the IdU administration (Fig. 3A). The SVZ contains long-term label-retaining cells that were IdU-positive (IdU+) (Fig. 3B). Most IdU+ cells continued proliferating and had incorporated both IdU and CIdU (IdU+CIdU+). These cells are likely to be the self-renewing neural stem cells [37]. Interestingly, 98±4% (n = 3) of the IdU+CIdU+ cells were TLX-positive (Fig. 3B), suggesting that vast majority of the self-renewing neural stem cells are TLX-expressing cells in the SVZ of adult brains. Only a very small fraction of IdU+ cells were CIdU-. In contrast, the triple labeled cells (IdU+CIdU+TLX+) comprised 14±3% (n = 3) of the total TLX-positive cells in the SVZ (Fig. 3B). The majority of the TLX-positive cells were not labeled by IdU or CIdU (Fig. 3B). This result, together with the observation that most of the TLX-positive cells were also positive for the neural stem cell marker nestin and GFAP (Fig. 1A, Fig. S1), suggests that the majority of TLX-positive cells are the relatively quiescent neural stem cells. Together, these results indicate that almost all of the self-renewing neural stem cells are TLX-expressing cells and that the TLX-positive neural stem cells in the SVZ include both the slowly dividing neural stem cells and the relatively quiescent neural stem cells.

Next we studied self-renewal in the SVZ of adult brains by analyzing Ki67/IdU/CIdU triple staining using animals treated with IdU and CIdU in the same scheme as described in the above (Fig. 3A). Animals were sacrificed two weeks after the last CIdU injection. The two-week survival should allow most migrating IdU+CIdU+ neuroblasts to be cleared from the SVZ to go to the olfactory bulb. Therefore, the IdU+CIdU+ cells that remained in the SVZ should be the self-renewing cells [37]. There were 78±6% (n = 3) of IdU+CIdU+ cells that were Ki67+ (Fig. 3C). However, almost all of the triple-labeled cells (IdU+CIdU+Ki67+) had very weak Ki67 staining (Fig. 3C). Among the Ki67-labeled cells, there were 34±14% (n = 3) of them were IdU+CIdU+ self-renewing neural stem cells (Fig. 3C).

### Decreased BrdU label-retaining and increased GFAP signals in TLX−/− SVZ

**Figure 4:**
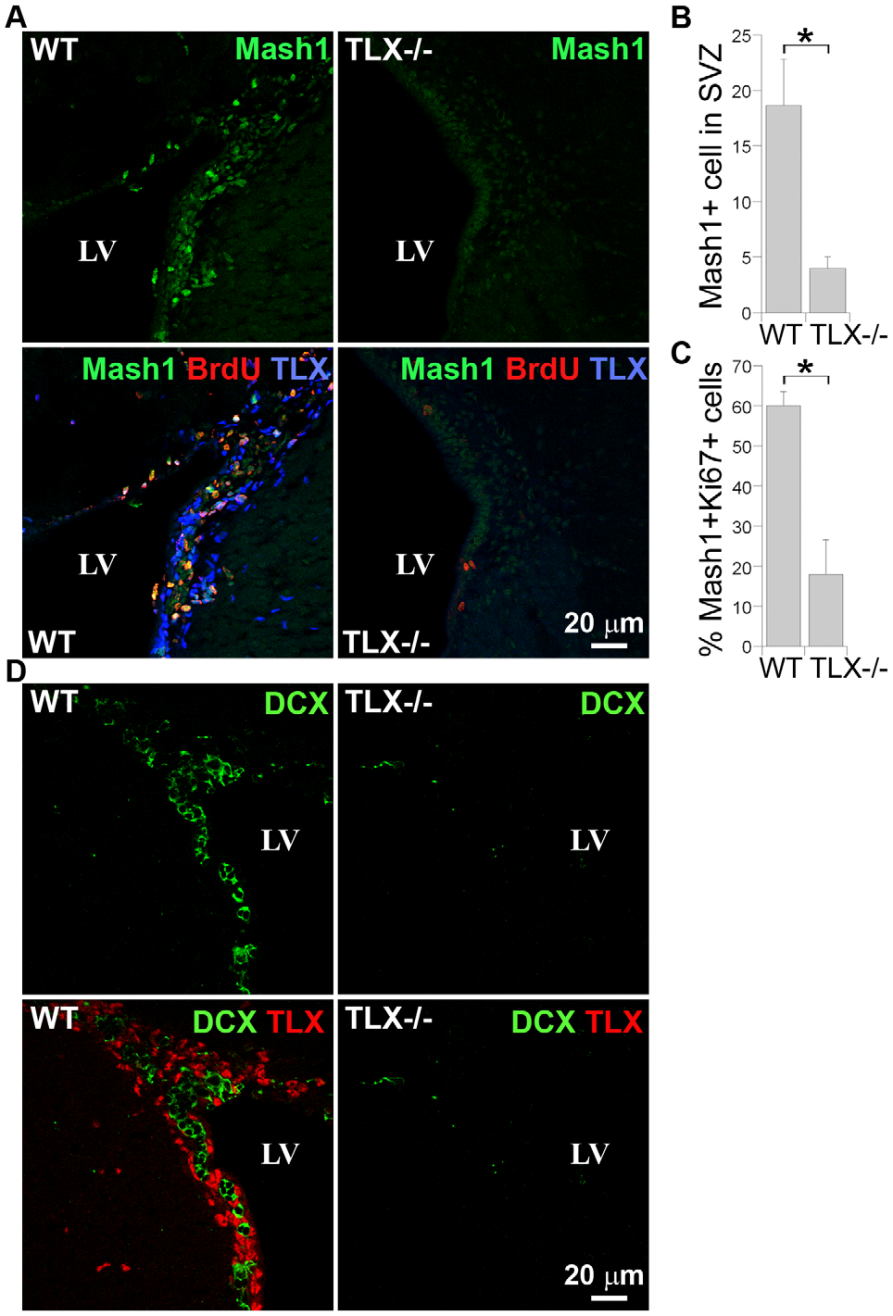
Reduced neural progenitor populations in the SVZ of TLX−/− brains. **A.** short-term (6 hr pulse) BrdU labeling along with Mash1 and TLX staining in the SVZ of wild type (WT) and TLX−/− brains. The top panels show Mash1 single staining and the bottom panels show merged images of Mash1, brdU and TLX triple staining. B. Quantification of Mash1+ cells in the SVZ of WT and TLX−/− brains. Data are represented as means ± s.d. *p<0.001 by Student's t-test. C. Quantification of Mash1+Ki67+ cells from Mash1+ cells in the SVZ of WT and TLX−/− brains. Data are represented as means ± s.d. *p<0.001 by Student's t-test. **D.** DCX and TLX staining in the SVZ of WT and TLX−/− brains. The top panels show DCX single staining and the bottom panels show merged images of DCX and TLX double staining. Scale bar, 20 µm for all panels.

We took advantage of the TLX knockout mouse model to examine the effect of how a loss of TLX expression affects the composition of the different cell types found in the SVZ of adult brains. Short-term BrdU labeling (a 6 hr BrdU pulse) showed a dramatic reduction in the number of rapidly dividing neural progenitor cells in the SVZ of TLX−/− brains, indicated by a decrease in 6 hr pulsed BrdU-labeling cells (Fig. 4A), which labels both type C and type A cells. Indeed, Mash1 and BrdU double staining revealed a considerable reduction in the number of BrdU+Mash1+ (type C) cells in the SVZ of TLX−/− brains (Fig. 4A). Quantification of the total numbers of Mash1-positive cells revealed a significant decrease in the SVZ of TLX−/− brains, compared to that in the SVZ of wild type brains (Fig. 4B). Furthermore, the proportion of the Mash1-positive cells that are proliferating (Ki67-positive) was also substantially reduced in the SVZ of TLX−/− brains (Fig. 4C). DCX staining revealed that DCX+ type A cells in the SVZ of TLX−/− brains were dramatically decreased as well (Fig. 4D).

**Figure 5:**
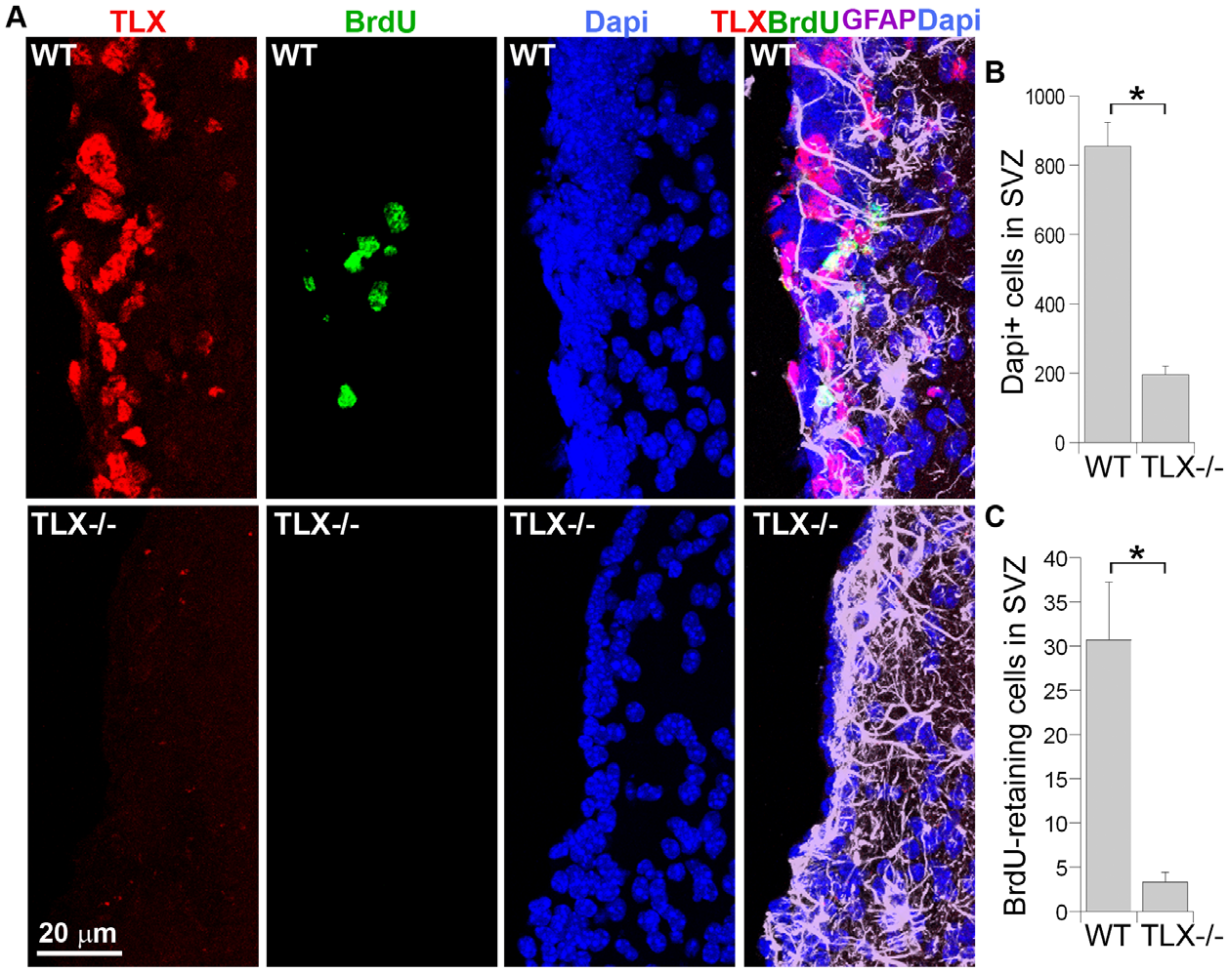
Reduced BrdU label-retaining cells and increased GFAP-positive cells in the SVZ of TLX−/− brains. **A.** There are reduced numbers of total cells and BrdU label-retaining cells in the SVZ of TLX−/− brains as revealed by Dapi staining (blue) and BrdU label (green) -retaining, and increased GFAP-positive cells as revealed by GFPA staining (purple). Both wild type (WT) and the TLX−/− mice were treated with BrdU once daily for 1 week, followed by 4 week survival. **B.** Quantification of Dapi-positive cells in the SVZ of wild type (WT) and TLX−/− brains. *p = 0.0015 by Student's t-test, n = 3. **C.** Quantification of BrdU label-retaining cells in the SVZ of WT and TLX−/− brains. *p = 0.019 by Student's t-test, n = 3. Error bars are standard deviation of the mean. Scale bar, 20 µm for all panels.

The total cell population in the SVZ, as revealed by nuclear Dapi staining, reduced dramatically in TLX−/− brains, compared to that in wild type brains (Fig. 5A, B). Only one or two layers of cells remained in the SVZ of TLX−/− brains, but there were about five to seven layers of cells in the SVZ of wild type brains (Fig. 5A). Dramatically reduced numbers of BrdU label-retaining cells were also observed in the SVZ of TLX−/− brains (Fig. 5A, C), suggesting that loss of TLX expression substantially reduced neural stem cell population and this result may explain the decreased numbers of neural progenitors (Fig. 4) and the hypocellularity (Fig. 5A, B) observed in the SVZ of TLX−/− brains.

**Figure 6:**
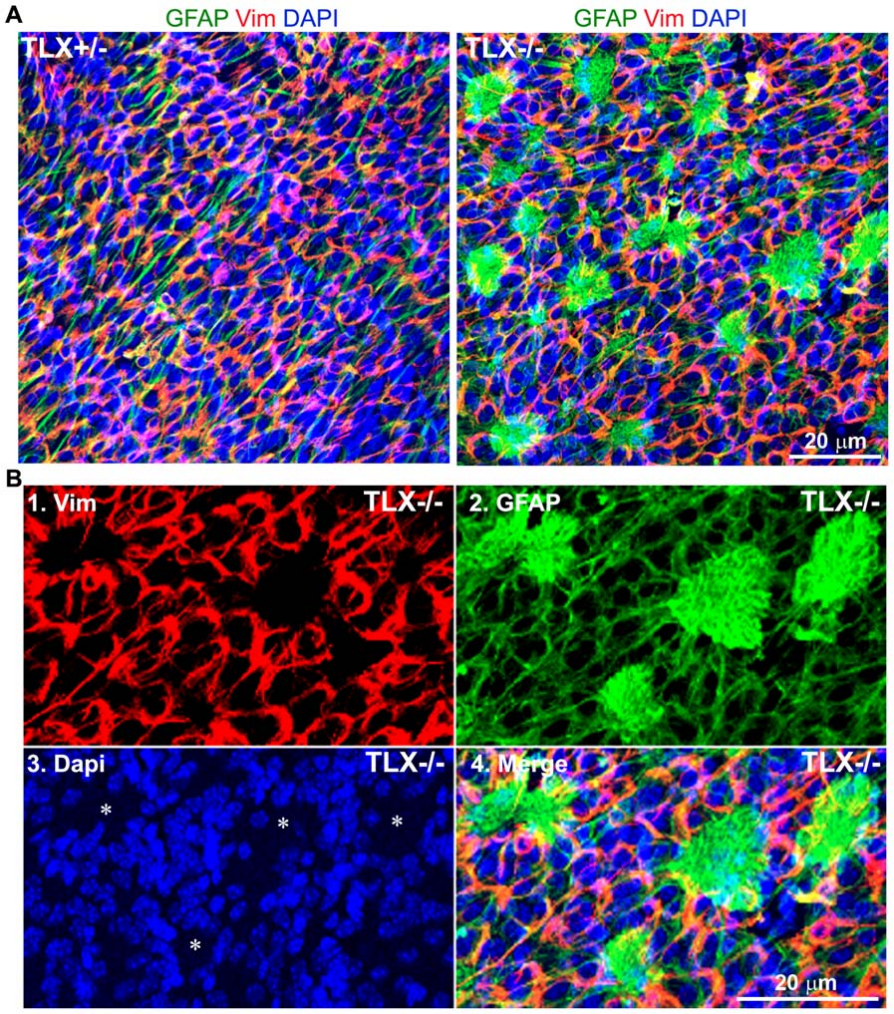
Whole mount staining revealed increased GFAP staining and scar-like GFAP-positive signals in the SVZ of TLX−/− brains. **A.** GFAP staining in the SVZ of TLX+/− and TLX−/− brains. Dapi and Vimentin (vim) staining was included as counter staining. **B.** Images of higher magnification of vimentin (1), GFAP(2), Dapi (3), and merged (4) staining in the SVZ of TLX−/− brains. Scale bar, 20 µm for all panels. Loss of Dapi staining in the Scar-like GFAP+ foci was indicated by asterisks.

Confocal images taken at the surface of whole mounts of the lateral wall of the lateral ventricles revealed intensive GFAP staining in the SVZ of TLX−/− brains (Fig. 6A). The GFAP staining was much stronger in the SVZ of TLX−/− brains, compared to WT and TLX+/− brains (Fig. 5A, 6A). Some of these GFAP signals formed scar-like structures that projected into the lateral ventricles in the TLX−/− SVZ (Fig. 6A, B). The scar-like GFAP+ structures in the TLX−/− SVZ are associated with apparent cell loss, as revealed by loss of Dapi staining in these loci (Fig. 6B, indicated by asterisks). Together, these results indicate that loss of TLX expression resulted in the depletion of neural stem cell pools in the SVZ of adult brains and hyper-intensive GFAP expression, presumably due to gliosis.

## Discussion

This study provided the first evidence of endogenous TLX expression in the SVZ of adult mouse brains by immunostaining with a TLX-specific antibody. TLX immunostaining was performed to determine the cellular identity of the TLX-expressing cells in the SVZ regions of adult mouse brains. Different strategies of thymidine analog incorporation in the wild type and TLX-homozygous mice were used to determine the proliferative status of the TLX-expressing cells.

The adult SVZ contains the slowly dividing or relatively quiescent type B neural stem cells and the rapidly dividing progenitors, including the transit-amplifying type C cells and type A neuroblasts [20], [21]. Our data indicated that TLX is mainly expressed in the slowly dividing or relatively quiescent type B neural stem cells and some rapidly dividing type C cells in the SVZ region of adult brains. Most of the BrdU label-retaining neural stem cells are TLX-positive cells. In addition to label-retaining neural stem cells, TLX is also expressed in rapidly dividing type C cells with Mash1 expression and short-term BrdU incorporation.

A previous study has shown that TLX is expressed exclusively in type B cells but not in type C cells in the SVZ of adult mouse brains, using a CreER^T2^ reporter under the control of TLX gene regulatory sequences [14]. The discrepancy of the present study with the previous observation could be explained by two possible reasons. One is that the TLX antibody is used to probe endogenous TLX expression in this study, whereas a CreER^T2^ reporter was used to determine TLX expression in the previous study. Secondly, we used Mash1 to label type C cells in this study, while epidermal growth factor receptor (EGFR) was used to label type C cells in the previous study [14]. Our observation that TLX is expressed in rapidly dividing type C cells is further supported by a recent report that TLX is expressed in EGFR^high^ transit amplifying cells [10].

In this study, we also took advantage of a double tymidine analog labeling approach to investigate the proliferative state of the TLX-expressing cells in the SVZ of adult brains. The thymidine analogs IdU and CIdU have advantages over BrdU labeling alone. Both IdU and CIdU can be injected into the same animal, therefore cells produced at different time points can be simultaneously assessed [29]–[31]. Double labeling of IdU and CIdU allowed us to establish if the TLX-positive cells that were label-retaining eventually re-entered cell cycle [35]. Using the triple labeling of IdU, CldU and TLX, we demonstrated that most of the TLX+ cells that incorporated the first thymidine analog (IdU+) in the SVZ also had the incorporation of the second thymidine analog (CIdU+). Very few TLX+IdU+ cells were detected as CldU-negative. These data imply that once the TLX+ slowly dividing cells are activated to enter the cell cycle, they keep self-renewing with slow division until they give rise to the rapidly dividing type C cells. Moreover, about 98% of the IdU+CIdU+ cells are TLX-positive cells, suggesting that the TLX-expressing cells constitute the majority of the self-renewing neural stem cells in the SVZ of adult brains.

Interestingly, a large number of TLX-expressing cells that were not labeled by either IdU or CIdU were detected in the SVZ of adult brains. Similarly, a big portion of the TLX-expressing cells did not label with either short-term or long-tem BrdU labeling in the SVZ. These observations suggest the presence of a population of TLX-positive cells that are relatively quiescent and arrested in the cell cycle. Quiescence has been shown to be important for the maintenance of the regenerative potential of adult neurogenic regions, presumably by functioning as a protective mechanism to counter stem cell exhaustion [38]. The expression of TLX in the quiescent neural stem cells in the SVZ of adult brains suggests an important role for TLX in maintaining the germinal reservoirs during adult neurogenesis.

It has been shown that the slowly dividing, BrdU label-retaining cells correspond to neural stem cells in the adult brain [33]. GFAP has also been shown to be a marker of the label-retaining type B neural stem cells in the SVZ of adult brains [21], [39]. Interestingly, we show here that loss of TLX expression led to dramatically reduced BrdU label-retaining cells. However, substantially enhanced GFAP immunostaining was detected in the SVZ of TLX−/− brains. The GFAP+ cells in the SVZ regions of TLX−/− brains exhibit extensive process networks. Whole mount images of the SVZ revealed that some of the GFAP+ processes form scar-like structure in the SVZ of TLX−/− brains. These GFAP+ cells lose neural stem cell properties and are no longer BrdU label-retaining. These results suggest that TLX is essential to maintain the self-renewing neural stem cells in the SVZ of adult mouse brains and that the GFAP+ cells in the VZ/SVZ lose neural stem cell property upon loss of TLX expression.

## Materials and Methods

### Ethics statement

All research animals are maintained in accordance with the NIH Guide for the Care and Use of Laboratory Animals and used in compliance with federal and institutional regulations. Brain tissues were harvested from wild type, TLX-heterozygous, and TLX-homozygous mice under the IACUC protocol 03038 approved by the City of Hope Institutional Animal Care and Use Committee.

### Immunohistochemistry

TLX-heterozygous and homozygous mice were described in our previous study [3]. Wild type, TLX-heterozygous, and TLX-homozygous adult mice were perfused with 4% paraformaldehyde in 0.1 M PBS, pH 7.4. Coronal sections of frozen brains were processed for immunostaining. Specifically, brain sections were incubated with blocking solution containing 5% normal donkey serum and 0.1% Triton X-100 in PBS for 1 hr, and then incubated with primary antibodies in the blocking solution at 4°C overnight. Subsequently, sections were washed in PBS and then incubated with secondary antibodies in PBS with 0.1% Triton X-100 for about 1.5 hr at room temperature. The sections were washed in PBS and mounted in fluorescent mounting medium. For immunostaining of thymidine analogs and Mash1, tissues were first incubated in 2 N HCL at 37°C for 30 minutes and then rinsed in PBS. After rinsing, tissues were immunostained as described above. Confocal microscopy was performed using a Zeiss LSM 510 Meta Inverted 2 Photon or LSM 510 Upright 2 Photon (Carl Zeiss, Germany).

### Antibodies

Primary antibodies used include rabbit anti-TLX (1∶1,000) [18]; mouse anti-glial fibrillary acidic protein (GFAP) (1∶1,000, Sigma) or guinea pig anti-GFAP (1∶1,000, Advanced ImmunoChemical); rat anti-5′-bromo-2′-deoxyuridine (BrdU) (1∶2,000, Accu-Specs, for Brdu and CIdU staining) or mouse anti-BrdU (1∶2,000, Becton Dickinson Immunocytometry Systems, for IdU staining); mouse anti-Mash1 (1∶100, BD PharMingen); goat anti-DCX (1∶300, Santa Cruz Biotechnology); and mouse IgM anti-vimentin, (1∶2000, Sigma). Secondary antibodies used include FITC, Cy3 or Cy5-conjugated donkey anti-mouse, rabbit, rat, goat IgG, donkey anti-mouse IgM (Jackson ImmunoResearch), or Alexa Fluor 488, Alexa Fluor 555-conjugated donkey anti-mouse, rabbit or goat IgG (Molecular Probes).

### Thymidine analog incorporation

To label proliferative cells, we treated mice with BrdU at 50 µg/g body weight in three strategies. For short-term BrdU labeling, animals were injected with BrdU and sacrificed 6 hr later to chase the rapidly dividing cells (6 hr BrdU chase). Alternatively, mice were injected with BrdU once daily for one week and sacrificed the next day after the last dose of BrdU to chase most of the dividing cells. For long-term BrdU labeling, mice were injected with BrdU once daily for one week and sacrificed 4 weeks later to chase the slowly dividing cells (BrdU label-retaining cells). For double IdU and CIdU labeling to chase the continuously slowly dividing cells, mice were treated with IdU at 0.1% in drinking water for 2 weeks. Ten days later, mice were injected with CldU at 50 µg/g body weight once daily for 5 days. The treated animals were allowed to survive for 2 weeks and then sacrificed and processed for immunostaining analysis.

## Supporting Information

Fig. S1 **Expression of TLX in GFAP-positive type B neural stem cells in the SVZ of mouse brains.** **A.** A single optical scanning image of TLX-GFAP staining. B. A merged image from a z-series scanning of the same staining. Nuclei DAPI staining was shown in blue. LV stands for lateral ventricles.
(TIF)

Fig. S2 **Orthogonal images and multiple single plane images of co-staining of TLX, Mash1, and BrdU.** **A.** Orthogonal images of TLX, Mash1, and BrdU co-staining. The 1^st^, 2^nd^, 3^rd^, and 4^th^ cells (from the top) in Fig. 1B are shown in orthogonal planes. **B.** Multiple single plane images of TLX, Mash1, and BrdU co-staining. LV stands for lateral ventricles.
(TIF)

Fig. S3 **Orthogonal images of TLX-BrdU co-staining.** An example of the TLX-BrdU double-stained cells in long-term BrdU labeling (1) and short-term BrdU labeling (2) is shown in orthogonal planes. LV stands for lateral ventricles.
(TIF)
