# Supplementary figures and images for: Characterization of TLX Expression in Neural Stem Cells and Progenitor Cells in Adult Brains
Source: PLoS One. 2012 Aug 30;7(8):e43324. doi: 10.1371/journal.pone.0043324 (PMC3431389; doi:10.1371/journal.pone.0043324)

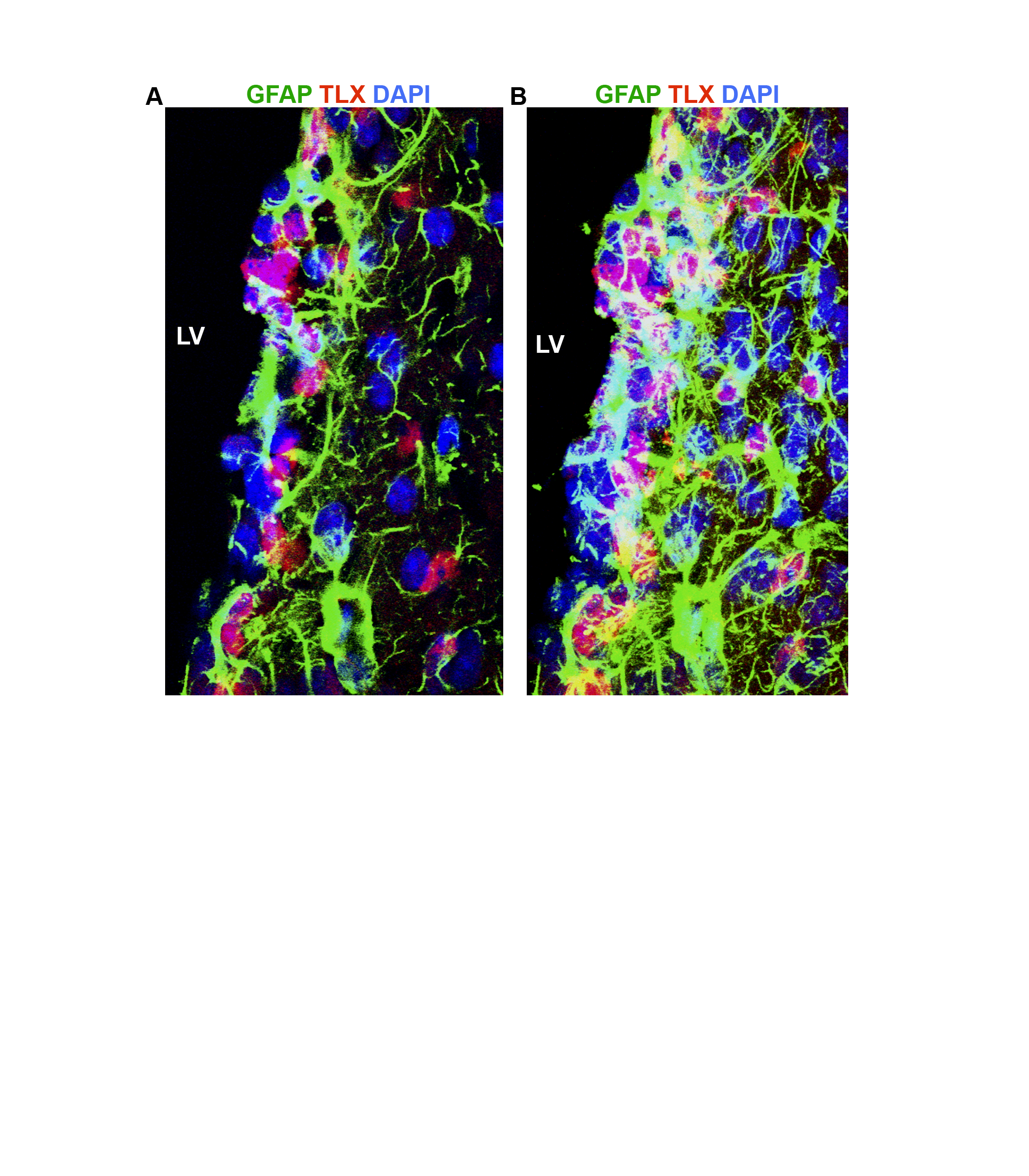

Supplement: Fig. S1 — Expression of TLX in GFAP-positive type B neural stem cells in the SVZ of mouse brains. A. A single optical scanning image of TLX-GFAP staining. B. A merged image from a z-series scanning of the same staining. Nuclei DAPI staining was shown in blue. LV stands for lateral ventricles. (TIF) [file pone.0043324.s001.tif]

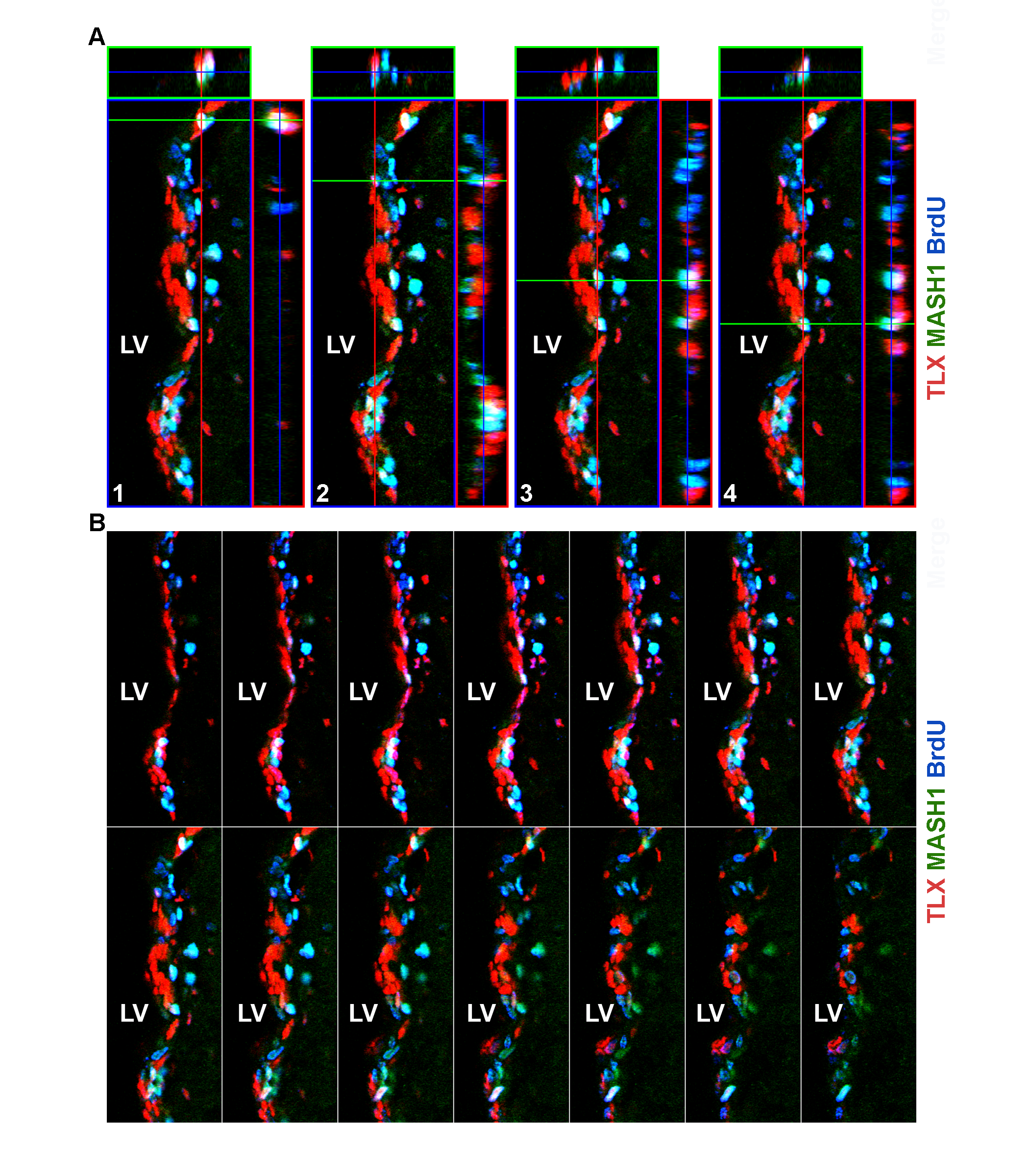

Supplement: Fig. S2 — Orthogonal images and multiple single plane images of co-staining of TLX, Mash1, and BrdU. A. Orthogonal images of TLX, Mash1, and BrdU co-staining. The 1st, 2nd, 3rd, and 4th cells (from the top) in Fig. 1B are shown in orthogonal planes. B. Multiple single plane images of TLX, Mash1, and BrdU co-staining. LV stands for lateral ventricles. (TIF) [file pone.0043324.s002.tif]

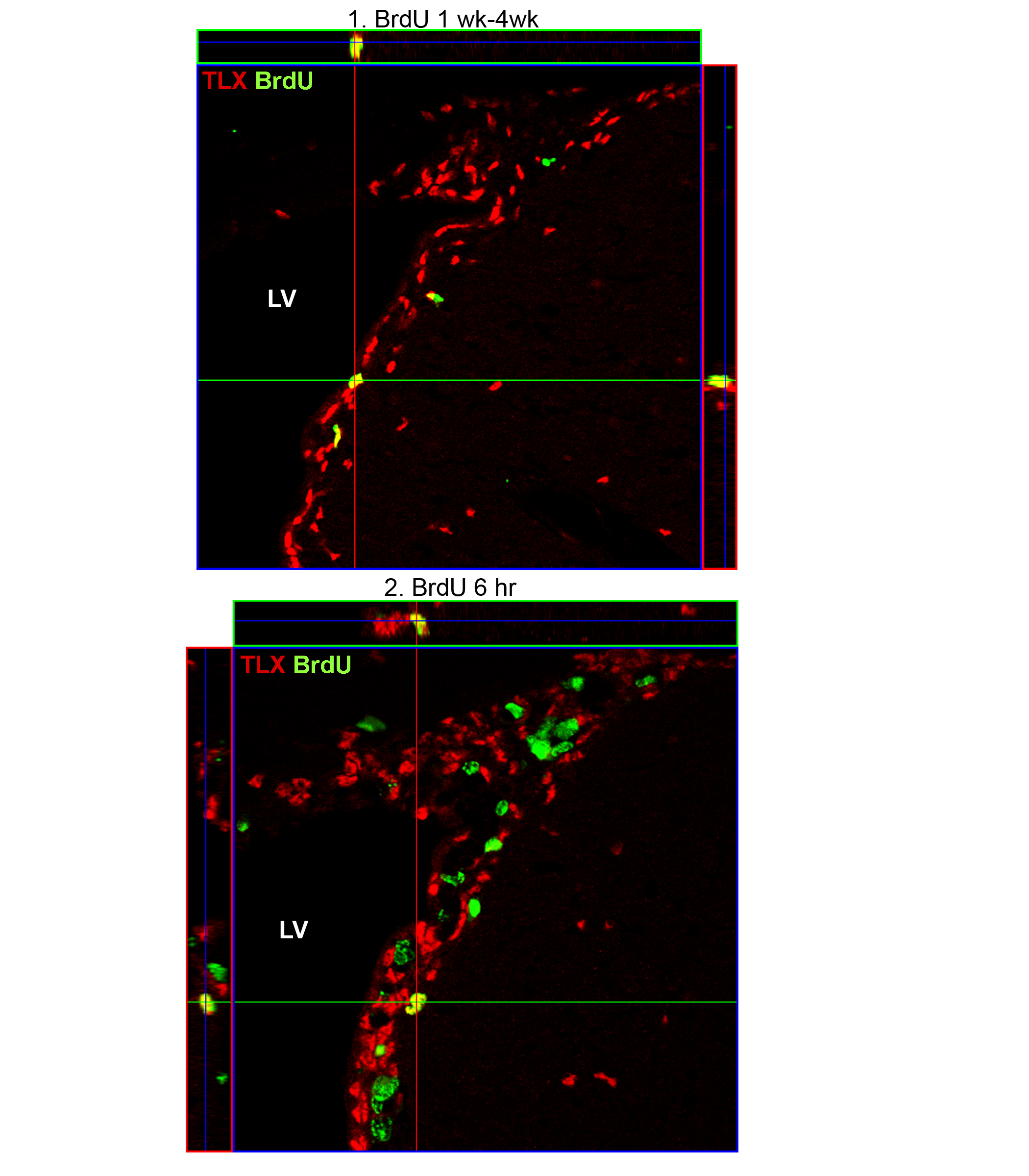

Supplement: Fig. S3 — Orthogonal images of TLX-BrdU co-staining. An example of the TLX-BrdU double-stained cells in long-term BrdU labeling (1) and short-term BrdU labeling (2) is shown in orthogonal planes. LV stands for lateral ventricles. (TIF) [file pone.0043324.s003.tif]
